# Identification of Quantitative Trait Loci (QTL) for Canine Hip Dysplasia and Canine Elbow Dysplasia in Bernese Mountain Dogs

**DOI:** 10.1371/journal.pone.0049782

**Published:** 2012-11-26

**Authors:** Sophia Pfahler, Ottmar Distl

**Affiliations:** Institute for Animal Breeding and Genetics, University of Veterinary Medicine Hannover, Hannover, Germany; University of Queensland, Australia

## Abstract

A genome-wide association study for canine hip dysplasia (CHD) and canine elbow dysplasia (CED) using the Illumina canine high density bead chip had been performed for 174 Bernese mountain dogs. General and mixed linear model analysis identified two different regions with single nucleotide polymorphisms (SNPs) on dog chromosome (CFA) 14 significantly associated with CHD and a further significantly CHD-associated region on CFA37. For CED, four SNPs on CFA11 and 27 were significantly associated. The identified SNPs of four associated regions included nearby candidate genes. These possible positional candidates were the genes *PON2* on CFA14 and *FN1* on CFA37 for CHD and the genes *LMNB1* on CFA11 and *WNT10B* on CFA27 for CED.

## Introduction

Hip dysplasia (CHD) and elbow dysplasia (CED) are common orthopedic conditions of the dog. These diseases affect dogs of all breeds with different prevalences. CHD is characterized by instability and subluxation or luxation of hips causing erosion of the articular cartilage and synovitis. CED results in osteoarthrotic changes, incongruity of the joint and lameness. Radiographic diagnosis of CED includes osteoarthrotic changes and the primary lesions incongruity of the elbow joint, fragmented medial coronoid process, osteochondrosis dissecans of the trochlea humeri and an ununited anconeal process. Genetic and environmental factors influence the expression of CHD and CED [1]–[4]. The Bernese mountain dogs (BMD) are affected by both diseases. Prevalences between 23 and 32% were reported for CHD in this breed, with the highest prevalence of CHD-affected dogs in Germany [5]–[8]. Among 20 and 37% dogs, CED was found in the various European BMD populations [5]–[7], [9]–[10]. Heritabilities were estimated at 0.26–0.42 for CHD and at 0.17–0.38 for CED in BMD populations from Finland, Sweden and Germany [5], [7], [10].

BMD belong to the large dog breeds officially recognized by the FCI (Fédération Cynologique Internationale) under the FCI-standard number 45. This breed has an ancestral origin in the area of the Canton Berne in the Switzerland and after breed foundation as early as 1907, this breed became worldwide popular as a companion, family and multi-purpose dog. Originally, BMD were used for cattle driving and as guard and draught dogs on farms in Switzerland and South Germany. Like the other three Sennenhund breeds, the BMD has a distinctive tricolored hair coat, but is the only Sennenhund breed with a long haircoat (http://www.nmbe.ch/research/vertebrates/research/kynologie/swiss-dog-breeds/bernese-mountain-dog).

Quantitative trait loci (QTL) controlling CHD were identified on 19 chromosomes using genome-wide microsatellite sets for linkage analysis in German shepherd dogs [11], on twelve chromosomes for a Labrador Greyhound crossbreeding pedigree [12]–[14] and on two chromosomes for Portuguese water dogs [15]–[16]. A genome-wide association study, using the Labrador Greyhound crossbreeding pedigree and six dog breeds, revealed four CHD-associated SNPs on three chromosomes and two osteoarthritis-associated SNPs on two chromosomes [14]. An association of a *fibrillin 2*-haplotype with hip osteoarthritis and an increased expression of *MIG-6* and *fibronectin* in osteoarthritic cartilage were shown in dogs [17]–[19].

The collagen genes *COL1A1, COL1A2, COL2A1, COL3A1, COL5A1, COL5A2, COL6A3, COL9A1, COL9A2, COL9A3, COL10A1, COL11A1, COL11A2*, and *COL24A1* were eliminated as candidates for fragmented coronoid process in Labrador retrievers [20].

The objectives of the present study were to identify SNPs associated with CHD and CED using the Illumina high density canine bead chip (Illumina, San Diego, CA, USA) for BMD. In the genomic regions identified through the genome-wide association study (GWAS), we determined positional candidate genes by their involvement in the human or murine development of cartilage and the pathophysiology of human osteoarthritis (OA).

## Results

**Figure 1:**
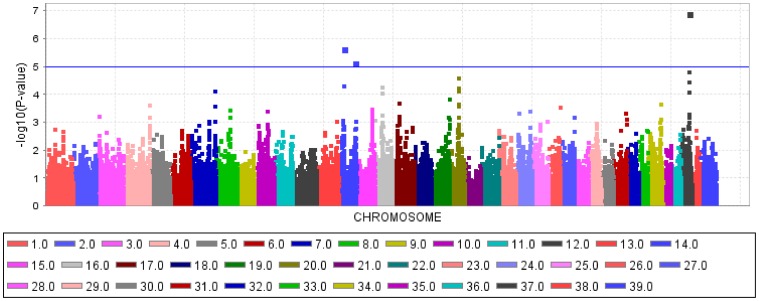
Manhattan plot of −log_10_P-values of the genome-wide association study for the canine hip dysplasia score in Bernese mountain dogs using a general model analysis. On the X-axis, the SNPs are given by dog chromosome number. The −log_10_P-values for each SNP genotype effect are plotted against the SNP position on each chromosome. Chromosomes are differentiated by colors. The color keys are given below the plot. The blue line indicates the threshold of the −log_10_P-values for genome-wide significance after correcting for multiple testing.

**Figure 2:**
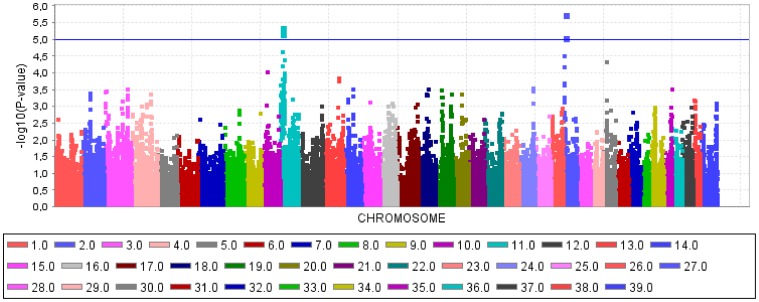
Manhattan plot of −log_10_P-values of the genome-wide association study for the canine elbow dysplasia score in Bernese mountain dogs using a general model analysis. On the X-axis, the SNPs are given by dog chromosome number. The −log_10_P-values for each SNP genotype effect are plotted against the SNP position on each chromosome. Chromosomes are differentiated by colors. The color keys are given below the plot. The blue line indicates the threshold of the −log_10_P-values for genome-wide significance after correcting for multiple testing.

**Figure 3:**
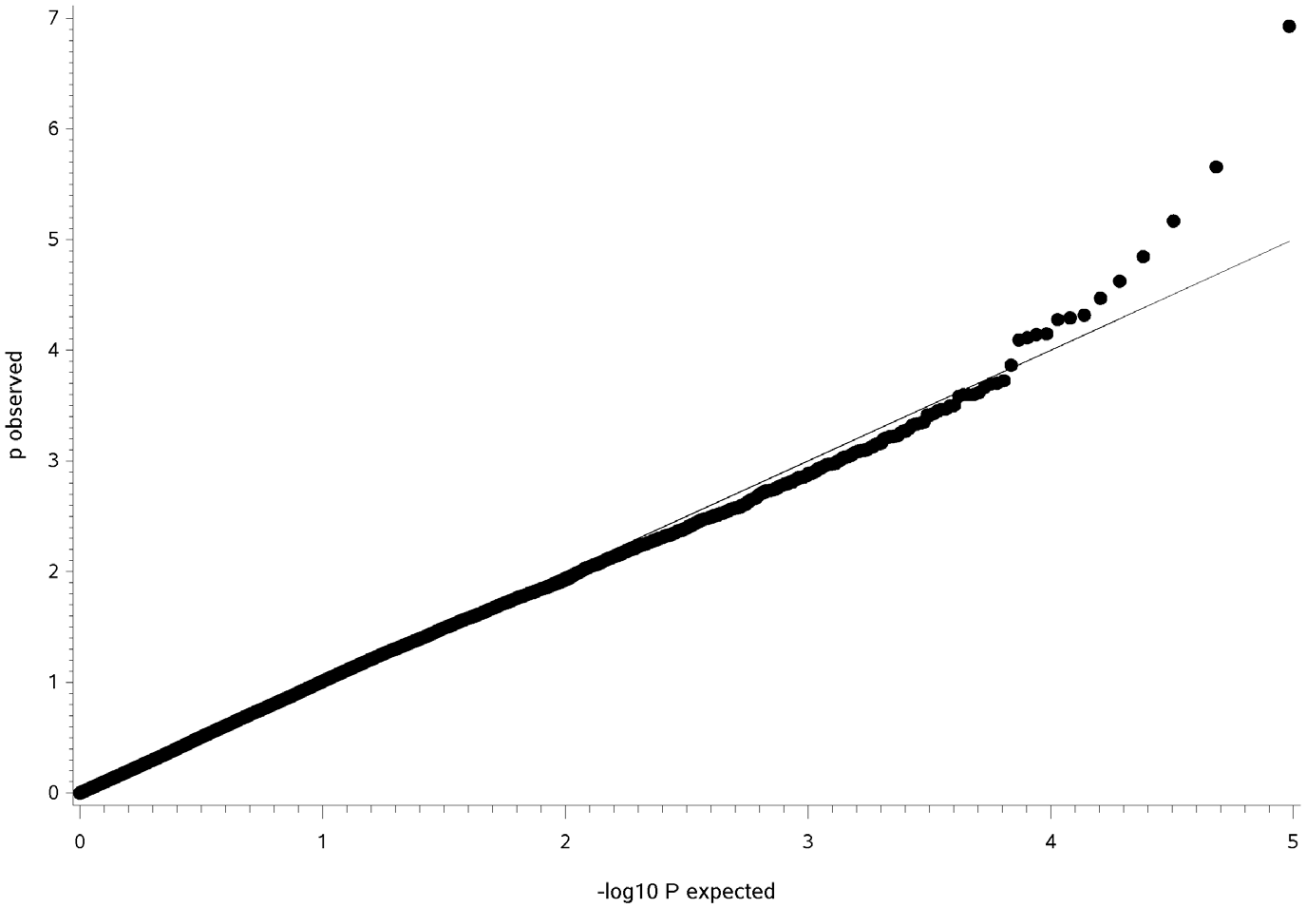
Q-Q-plot of expected −log_10_P-values versus observed−log_10_P-values from the general model analysis for canine hip dysplasia score in Bernese mountain dogs. Shown are all 96,412 SNPs included in the genome-wide association analysis with the grey line corresponding to the null hypothesis of no association.

**Figure 4:**
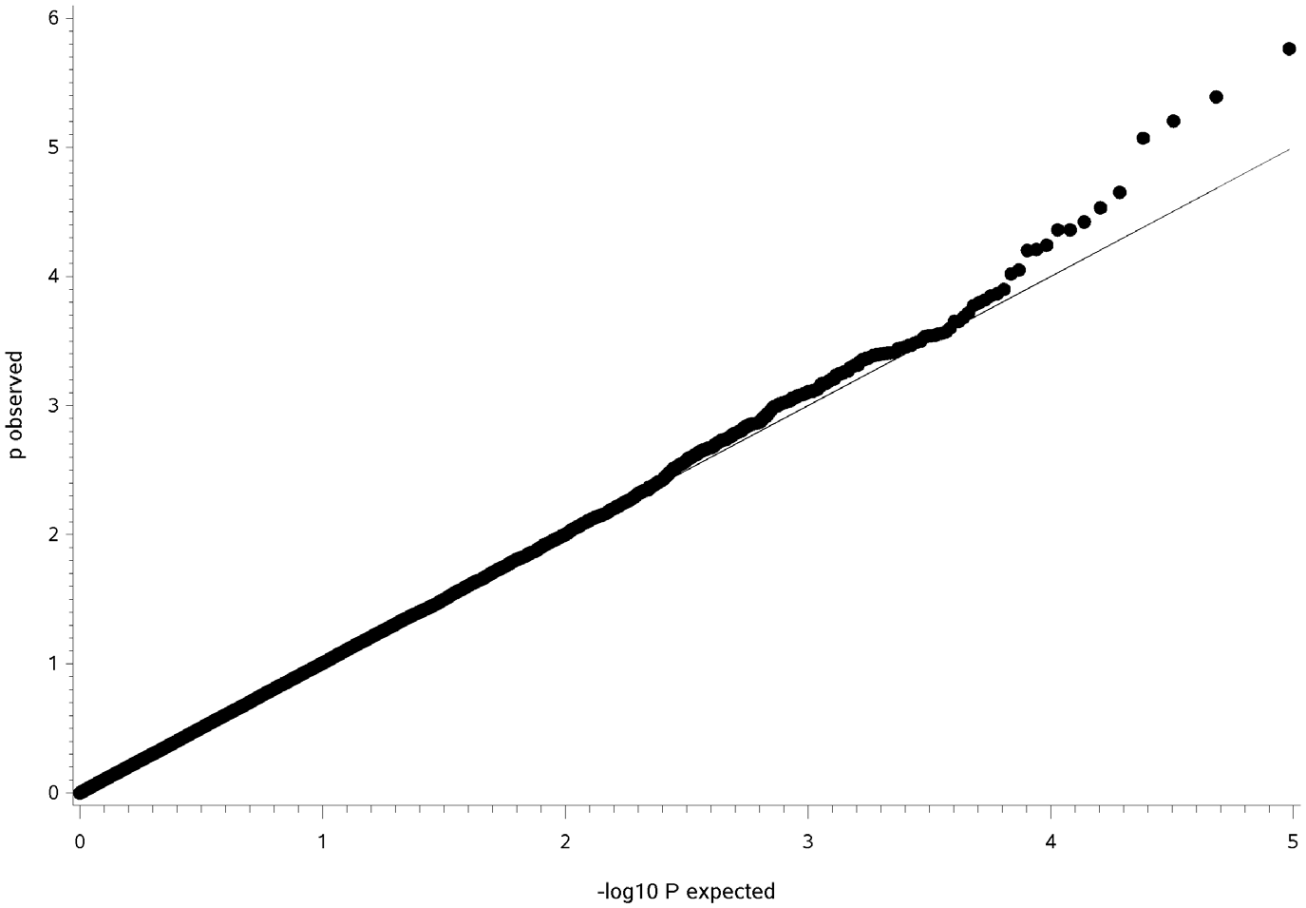
Q-Q-plot of expected −log_10_P-values versus observed−log_10_P-values from the general model analysis for canine elbow dysplasia score in Bernese mountain dogs. Shown are all 96,412 SNPs included in the genome-wide association analysis with the grey line corresponding to the null hypothesis of no association.

The GWAS using a general linear model (GLM) identified three QTL for CHD (Figure 1) and two QTL for CED (Figure 2) on four different chromosomes harbouring significantly associated SNPs. The quantile-quantile (Q–Q) plots illustrated that inflation due to stratification effects had been removed by the general linear models employed for CHD and CED (Figures 3, 4). We determined the threshold for significance at a −log_10_P-value of 4.97 which corresponds to a P-value of 0.05 after applying the Benjamini-Hochberg-FDR correction for multiple testing. The −log_10_P-values corrected for multiple testing were at 2.0 (BICF2P1089246 on CFA14), 1.51 (BICF2P1282232 on CFA14) and 3.27 (BICF2S23052396 on CFA37) for CHD and 1.55 (BICF2G630294653 on CFA11), 1.73 (BICF2G630294836 on CFA11), 1.41 (BICF2P1025413 on CFA27) and 2.11 (BICF2G630140058 on CFA27) for CED. Mixed linear model (MLM) analyses confirmed the associations of the same SNPs as found by the GLM analysis (Table S1).

In order to show the impact of the significantly associated SNPs on disease occurrence of CHD and CED, the variance explained by the significantly associated SNPs in the GLM and odds ratios (ORs) were estimated using sex as strata (Table 1). The ORs for the CHD-SNPs were at 2.08–3.45 and for the CED-SNPs at 2.94–3.21.

**Table 1:** Summary of results for the genome-wide association study using a general linear model analysis and sex-stratified case-control analysis for canine hip and elbow dysplasia in Bernese mountain dogs.

Trait	Position	SNP-ID	Minor	MAF	MAF_a_	MAF_u_	V_SNP_	OR	CI-L	CI-U	−log_10_P	Candidate	Distance to the nearest candidate		
CFA			allele									gene	gene in kb		
Canine hip dysplasia															
14	23,811,133	BICF2P1089246	C	0.32	0.46	0.29	0.14	2.08	1.22	3.57	5.66	*PON2*	183		
14	59,537,633	BICF2P1282232	T	0.15	0.29	0.11	0.12	3.08	1.63	5.85	5.17	*–*		–	
37	25,095,511	BICF2S23052396	A	0.12	0.25	0.09	0.15	3.45	1.75	6.67	6.93	*FN1*	361		
Canine elbow dysplasia															
11	18,913,755	BICF2G630294653	T	0.21	0.36	0.16	0.12	2.98	1.68	5.29	5.21	*LMNB1*	89		
11	19,114,139	BICF2G630294836	A	0.22	0.38	0.17	0.13	2.94	1.67	5.26	5.39	*LMNB1*	55		
27	7,848,483	BICF2P1025413	T	0.15	0.29	0.11	0.12	3.20	1.68	6.08	5.08	*WNT10B*	763		
27	7,956,078	BICF2G630140058	G	0.16	0.31	0.12	0.13	3.21	1.74	5.93	5.76	*WNT10B*	655		

The SNP-ID, the position on dog chromosome (CFA) in base pairs (bp), minor allele, minor allele frequency (MAF) for all, affected (MAF_a_) and unaffected (MAF_u_) dogs (controls), variance explained by the single SNPs (V_SNP_) and −log_10_P-values (−log_10_P) of the general model analysis are given. Odds ratios (OR) with 95% confidence intervals (CI) are from a sex-stratified case-control analysis for CHD- and CED-affected dogs. Positional candidate genes with their distance to the associated SNP are shown according to the dog genome assembly build 2.1.

**Figure 5:**
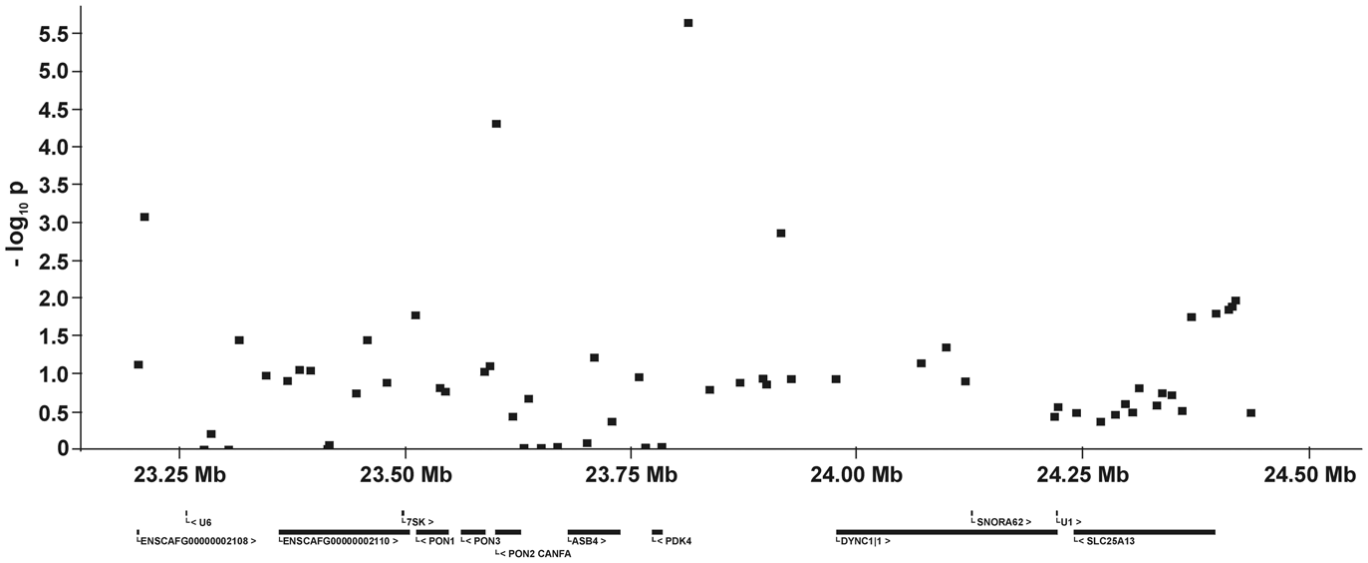
Genomic region with a significant association for canine hip dysplasia on dog chromosome (CFA) 14 at 23.25–24.5 Mb. The −log_10_P-values of all 62 SNPs in this region and the genes annotated according to the dog genome assembly build 2.1 are shown.

**Figure 6:**
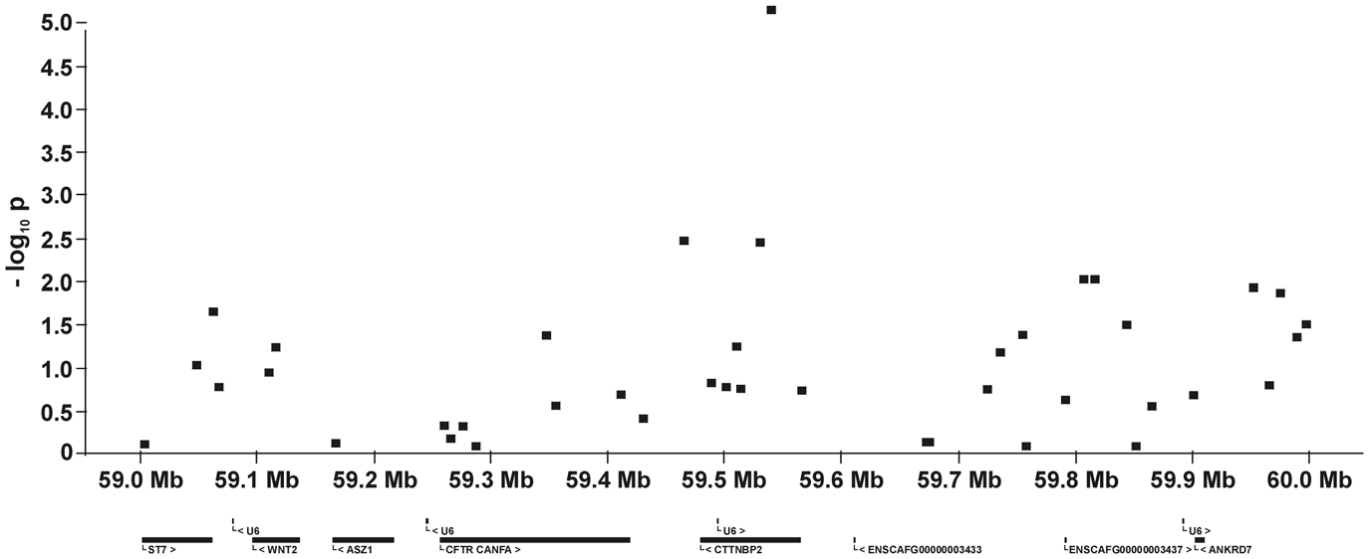
Genomic region with a significant association for canine hip dysplasia on dog chromosome (CFA) 14 at 59.5–60.0 Mb. The −log_10_P-values of all 41 SNPs in this region and the genes annotated according to the dog genome assembly build 2.1 are shown.

**Figure 7:**
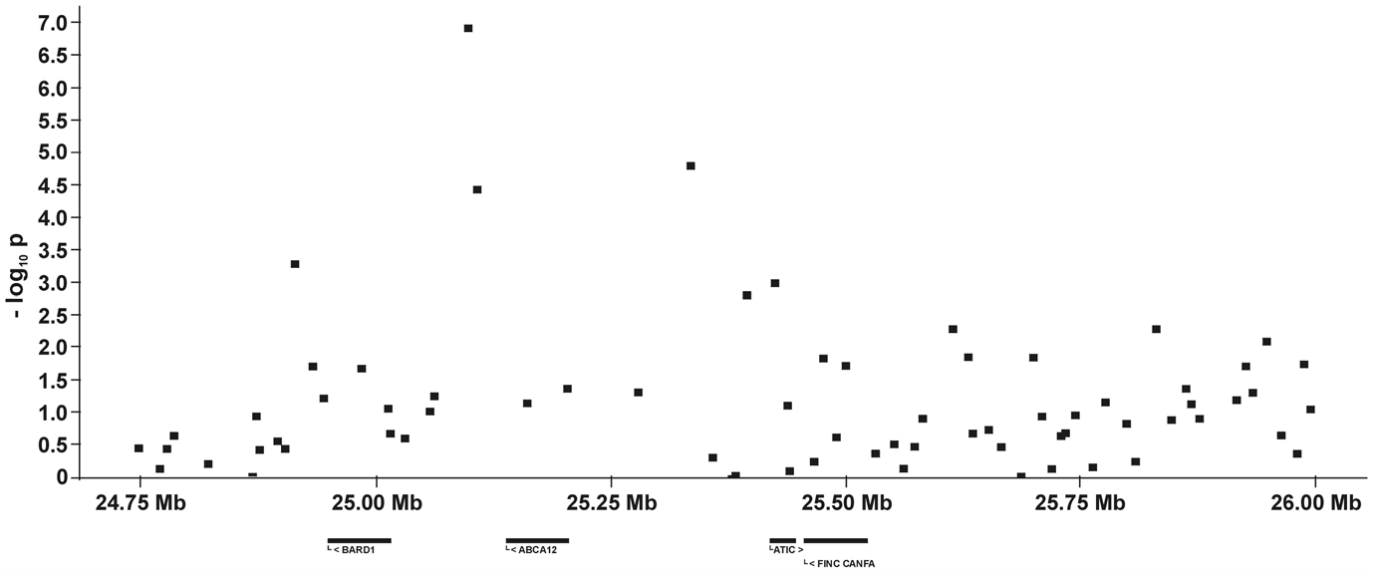
Genomic region with a significant association for canine hip dysplasia on dog chromosome (CFA) 37 at **24.75–26.0 Mb.** The −log_10_P-values of all 58 SNPs in this region and the genes annotated according to the dog genome assembly build 2.1 are shown.

**Figure 8:**
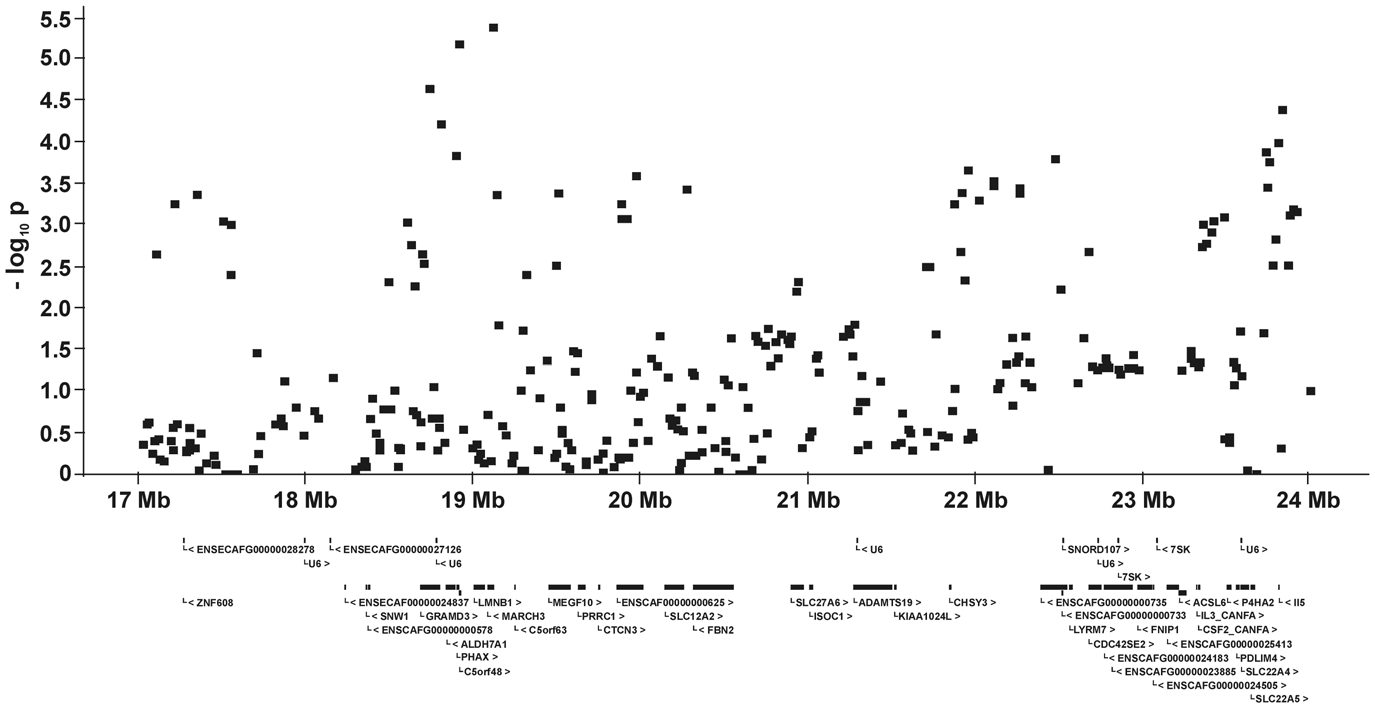
Genomic region with a significant association for canine elbow dysplasia on dog chromosome (CFA) 11 at 17.0–24.0 Mb. The −log_10_P-values of all 314 SNPs in this region and the genes annotated according to the dog genome assembly build 2.1 are shown.

**Figure 9:**
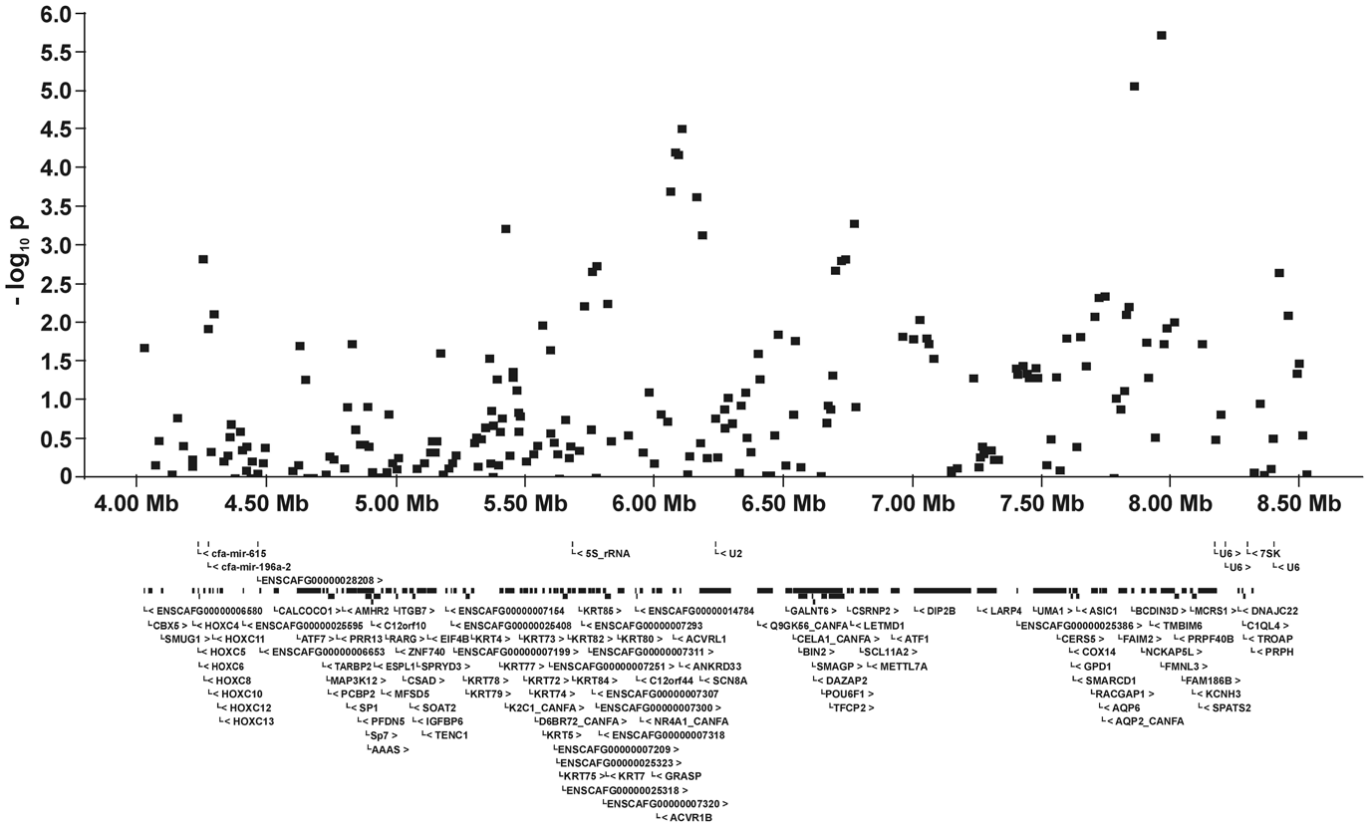
Genomic region with a significant association for canine elbow dysplasia on dog chromosome (CFA) 27 at 4.0–8.5 Mb. The −log_10_P-values of all 241 SNPs in this region and the genes annotated according to the dog genome assembly build 2.1 are shown.

For all five QTL and their flanking genomic regions, the −log_10_P-values and all genes annotated on the dog genome assembly build 2.1 are shown in Figures 5, 6, 7, 8, 9. Then, we analysed the haplotype structure and haplotype association for these genomic regions surrounding the significantly associated SNPs and containing CHD- or CED-SNPs with −log_10_P-values>2.0–3.0 using GLM analyses. For the haplotype association analyses, CHD and CED were treated as binary traits considering CHD- or CED-affected dogs as cases and dogs free from CHD and CED as controls. Haplotype blocks with significantly CHD- or CED-associated SNPs were identified within two of the five associated genomic regions (Table S2). On CFA37, there was one significantly CHD-associated haplotype at 25.0–25.2 Mb and this haplotype block contained the CHD-associated SNP BICF2S23052396. The chromosomal region on CFA11 at 17–24 Mb contained 44 haplotype blocks. In this region haplotypes from seven blocks were significantly associated with CED after applying permutation tests. The significantly CED-associated SNPs BICF2G630294653 and BICF2G630294836 on CFA11 were located within associated haplotype blocks at 18.80–19.07 and 19.11–19.13 Mb, respectively.

## Discussion

In the present study using the Illumina canine high density bead chip for 174 BMD, we identified three SNPs significantly associated with CHD on CFA14 and 37 and each two SNPs significantly associated with CED on CFA11 and 27. The results of the GWAS were consistent for the GLM and MLM even when the inbreeding coefficients and the first two to six principal components (PCA) were regarded as additional covariates. Two of the three significantly CHD-associated SNPs were located in intergenic regions. The CHD-associated SNP BICF2P1282232 was located within intron 9 of *CTTNBP2* (*cortactin-binding protein 2*). An obvious functional relationship of CTTNBP2 with CHD could not be found. An intron of the gene *MARCH3* on CFA11 harboured the CED-associated SNP BICF2G630294836. There was no obvious relation between this gene and the pathogenesis of CED. Similarly, the SNP BICF2G630294653 was located within intron 2 of *C5orf48* which encodes an uncharacterized protein. The further CED-associated SNPs on CFA27 were intergenic.

The human homologues of the genes *PON1*, *PON2* and *PON3* (*paraoxonase*) on CFA14 are part of the gamma-hexachlorocyclohexane degradation pathway that was associated with low bone mineral density of hips [21]. The *paraoxonase* 2 gene is nearest to the CHD-associated SNP BICF2P1089246 on CFA14, approximately 183 kb upstream of this SNP, and with this SNP located in the same haplotype block. The *protein phosphatase 1, regulatory subunit 9A* (*PPP1R9A*) gene that is imprinted in human and mouse was shown to be involved in the development of skeletal muscles [22]. A reduced muscle tonus can destabilize the hip joint and thereby possibly be a part of CHD pathogenesis. This gene is located approximately 609 kb upstream of the CHD-associated SNP on CFA14.

On CFA37, *FN1* (*fibronectin*), is located approximately 361 kb downstream of the CHD-associated SNP. FN1 is a component of the extracellular matrix in cartilage and an important function in matrix organization of cartilage had been suggested [23]. The fibronectin concentrations from canine femoral head cartilage with lesions were higher than from cartilage surrounding these regions [19]. In addition, in dogs with high risk to OA, fibronectin concentrations from explanted cartilage were higher in comparison to dogs with low OA risk [19].

The canine *lamin B1* (*LMNB1*) gene resides in a significantly CED-associated haplotype block at 18.80–19.07 Mb. Mice with a homozygous mutation causing the functional loss of *lamin B1* were nonviable because of respiratory problems and bone malformations. Growth and ossification of long bones were disturbed [24].

For the CED-QTL on CFA27, a candidate gene involved in chondrocyte metabolism, OA and osteoblast differentiation was found. The *wingless-type MMTV integration site family, member 10B* (*WNT10B*) gene approximately 655 kb downstream of the SNP BICF2G630140058 is associated with the bone mass and the bone mineral density in the human hip [25].

The SNP BICF2P1089246 on CFA14 is located within a CHD-QTL identified in a genome-wide linkage analysis using 261 microsatellites for German shepherd dogs [11]. The CHD-associated SNP on CFA37 at 25.0 Mb of the present study is on the same chromosome, but in far distance to the CHD-QTL found in the Labrador-Greyhound crossbreeding pedigree at 42.1–44.6 Mb [12], however, in the neighbourhood of a SNP associated with hip OA at 17.2 Mb detected in an across-breed GWAS [13]. As the FCI grading uses osteoarthrotic changes as criteria for the CHD-status, joint QTL for the CHD-status and OA may exist.

Genetic correlations among CHD and CED were close to zero in Swedish [7], at 0.26 in Finnish [10] and at 0.31 in German population genetic analyses for BMD [5]. No overlap was found between QTL for CHD and CED in the present GWAS and no common metabolic pathway was detected among genes located in CHD- and CED-associated chromosomal regions of the present GWAS. Thus, our study supports the assumption that CHD and CED are different genetic traits that may not share many common loci.

In summary, we mapped three loci on dog chromosomes (CFA) 14 and 37 significantly associated with CHD and two loci on CFA11 and 27 significantly associated with CED. The effects of the SNPs had ORs at 2.1–3.5 for disease occurrence. The SNPs with higher ORs had lower MAFs indicating that the alleles with higher risk to CHD and CED had a lower prevalence in BMD analysed here. Four of the five identified CHD- and CED-loci had nearby candidate genes.

## Materials and Methods

### Ethics Statement

All animal work has been conducted according to the national and international guidelines for animal welfare. The dogs in this study were included with the consent of their owners. The blood samples were collected by veterinarians during the routine examination for CHD and CED regarding principles of good veterinary practice. These diagnostic procedures had to be carried out anyway. All blood-sampling of dogs was done in veterinary practices for small animals by trained staff. CHD and CED examinations are compulsory for all dogs intended for breeding and these examinations were performed in veterinary clinics and practices according to the rules for hip screening of the FCI and the International Elbow Working Group (IEWG). Approval from the ethics committee was not obtained because blood sampling was done during a diagnostic veterinary procedure according to the German Animal Welfare Law (released on 05/18/2006, last changes on 12/09/2010).

### Animals

The sample genotyped included 174 purebred Bernese mountain dogs born in the years 1996 to 2010 (Table 2). In order to reduce the influence of the population structure for the GWAS, we chose only one descendent of each paternal grandsire from all dogs presented for X-raying of the hips and elbows from the birth year cohorts of 1996–2010.

**Table 2:** Number of Bernese mountain dogs genotyped on the Illumina high density beadchip.

CED-score	Female dogs	Male dogs	All dogs
Free	93	47	140
I	22	9	31
II	2	0	2
III	0	1	1
CHD-score			
A	87	49	136
B	4	1	5
C	26	7	33
Total	117	57	174

Number of dogs stratified by sex, canine hip dysplasia (CHD) and canine elbow dysplasia (CED) scores are given. Controls include dogs free from CHD and CED. Cases for CHD or CED are dogs classified with CHD-scores B or C or dogs classified with CED scores I, II or III. All dogs affected by CED, but three dogs were free from CHD.

All animals genotyped were screened for CHD according to the rules of the FCI and for CED according to the rules of the IEWG (http://www.vet-iewg.org/joomla/index.php/archive/23-2001-international-elbow-protocol-vancouver). The minimum age at the time of examination was twelve months for all dogs. A ventrodorsal X-ray of the hips and a flexed mediolateral projection of the elbow had to be taken by an accredited veterinarian with the dog in general anaesthesia. A breed specific expert evaluation report was provided for the X-rays scoring hip joints as normal (A), borderline (B), mild (C), moderate (D) or severe CHD (E) [26]–[27]. Elbows were categorized as free (ED 0) or mild to severe CED (ED I–ED III) based on the presence of primary lesions and secondary arthrotic changes. In Germany, presence of a primary elbow lesion (fragmented medial coronoid process, ununited anconeal process, osteochondrosis of the medial humeral condyle, radio-ulnar length discrepancy) leads to the classification of severe CED. Dogs classified as free form CHD and CED were used as controls and the other dogs as cases according to their scores for CHD or CED. The disease conditions for GWAS were treated as quantitative traits encoding CHD- and CED-unaffected dogs with one and the other conditions with 2–5 (CHD) or 2–4 (CED) for borderline (CHD) or mild (CED) to severe affection status.

### DNA Extraction

Extraction of genomic DNA from Na-EDTA blood samples was performed through a standard ethanol fractionation with concentrated sodiumchloride (6 M NaCl) and sodium dodecyl sulphate (10% SDS). Concentration of DNA was adjusted to 50 ng/µl in the samples used for genotyping.

### SNP Genotyping

Genotyping of the 174 BMD for the GWAS was done on the canine Illumina high density beadchip (Illumina) containing 173,662 SNPs. Quality criteria for further analyses were minor allele frequencies (MAF) >0.05, genotyping rate per SNP>0.90 and tests for Hardy-Weinberg equilibrium (p-value<0.000001). After filtering for quality criteria, 96,412 SNPs had been left for analysis. The mean genotyping rate per individual was 99 per cent.

### Statistical Analysis

The ALLELE procedure of SAS/Genetics, version 9.3 (SAS Institute, Cary, NC, USA) was used to calculate polymorphism information content, heterozygosity, allelic diversity, allele and genotype frequencies and χ^2^-tests for Hardy-Weinberg-Equilibrium for the SNPs genotyped. The GWAS was performed using both a general (GLM) and a mixed (MLM) linear model. CHD- and CED-scores were treated as quantitative traits (1 = CHD-A to 5 = CDH-E, 1 = ED free to 4 = ED III). The analyses were run using TASSEL, version 3.0.88 [28]. The GLM explained for sex effects and the respective SNP genotypes. The MLM included an additional random animal effect via the identity-by-state-kinship (IBS) matrix. The IBS matrix reflects the genomic relationship matrix among all individuals genotyped and captures the relatedness among animals as well as the cryptic family structure. Cryptic relatedness is due to distant relationships and therefore, may not be displayed through the pedigrees of the animals. Further GLM and MLM models with the inbreeding coefficients and the first two to six PCAs using as linear covariates had been run to test the robustness of the models. PCAs were parameterized to infer possible population stratification due to genetic ancestry. In order to avoid that blocks with long-range linkage disequilibria (LD) influence PCAs, we used a pruned set of SNPs in approximate linkage equilibria. Thus, PCAs and IBS-kinship coefficients were derived from a pruned set of 10,075 genome-wide and equidistantly distributed SNPs at pair-wise LD (r^2^) <0.3. The results for the extended models were consistent with the GLM and MLM analyses omitting PCAs and the inbreeding coefficient. Therefore, the results reported refer to GLM and MLM analyses with sex and the respective SNP genotypes as fixed effects and in the MLM with an additional random animal effect parameterized via the genomic relationship matrix. The reason for this consistency across the different models employed stems from the fact that PCAs, inbreeding coefficients and relatedness were rather equally distributed among affected and controls. We applied the Benjamini-Hochberg-FDR correction using the MULTIPLE TEST procedure of SAS, version 9.3, to determine the threshold for genome-wide significance.

Genotypic and allelic associations and allelic trends as well as ORs with their 95% confidence intervals for CHD- and CED-SNPs were calculated using the CASECONTROL procedure of SAS/Genetics and sex as strata.

We analysed the haplotype structure of the identified genomic regions and tested the associations of haplotype blocks using Haploview 4.0 [29]. CHD- and CED-scores had to be treated as binomial traits (CHD-A vs. CHD-B-E and ED free vs. ED I-ED III) in these analyses and a permutation rate of 10,000 was chosen.

## Supporting Information

Table S1 **Results for the genome-wide association study using a mixed linear model (MLM) analysis for canine hip dysplasia (CHD) and canine elbow dysplasia (CED) in Bernese mountain dogs.** The SNP-ID, the position on dog chromosome (CFA) in base pairs (bp) according to the dog genome assembly build 2.1, the variance explained by the single SNPs (V_SNP-MLM_) and −log_10_P-values (−log_10_P-MLM) of the MLM analysis are given.
(DOC)

Table S2 **Association tests for haplotype blocks using Haploview for canine hip dysplasia and canine elbow dysplasia.** The test statistics including χ^2^– and P-values (P) using 10,000 permutations for significantly associated haplotypes containing significantly associated SNPs from the general model analysis are shown. For these significantly associated SNPs, the SNP-ID and their chromosomal position are given.
(DOC)
