# Supplementary material for: Identification of Quantitative Trait Loci (QTL) for Canine Hip Dysplasia and Canine Elbow Dysplasia in Bernese Mountain Dogs
Source: PLoS One. 2012 Nov 26;7(11):e49782. doi: 10.1371/journal.pone.0049782 (PMC3506637; doi:10.1371/journal.pone.0049782)
Supplement: Table S1 — Results for the genome-wide association study using a mixed linear model (MLM) analysis for canine hip dysplasia (CHD) and canine elbow dysplasia (CED) in Bernese mountain dogs. The SNP-ID, the position on dog chromosome (CFA) in base pairs (bp) according to the dog genome assembly build 2.1, the variance explained by the single SNPs (VSNP-MLM) and −log10P-values (−log10P-MLM) of the MLM analysis are given. (DOC) [file pone.0049782.s001.doc]

**Table S1** **Results for the genome-wide association study using a mixed linear model (MLM) analysis for canine hip dysplasia (CHD) and canine elbow dysplasia (CED) in Bernese mountain dogs.** The SNP-ID, the position on dog chromosome (CFA) in base pairs (bp) according to the dog genome assembly build 2.1, the variance explained by the single SNPs (V_SNP-MLM_) and -log_10_P-values (-log_10_P-MLM) of the MLM analysis are given.

| CFA | Position | SNP-ID | Trait | V_SNP-MLM_ | -log_10_P-MLM |
| --- | --- | --- | --- | --- | --- |
| 14 | 23,811,133 | BICF2P1089246 | CHD | 0.13 | 5.31 |
| 14 | 59,537,633 | BICF2P1282232 | CHD | 0.12 | 4.59 |
| 37 | 25,095,511 | BICF2S23052396 | CHD | 0.16 | 6.26 |
| 11 | 18,913,755 | BICF2G630294653 | CED | 0.12 | 4.64 |
| 11 | 19,114,139 | BICF2G630294836 | CED | 0.12 | 4.85 |
| 27 | 7,848,483 | BICF2P1025413 | CED | 0.12 | 4.37 |
| 27 | 7,956,078 | BICF2G630140058 | CED | 0.12 | 5.05 |
