# Supplementary material for: Identification of Quantitative Trait Loci (QTL) for Canine Hip Dysplasia and Canine Elbow Dysplasia in Bernese Mountain Dogs
Source: PLoS One. 2012 Nov 26;7(11):e49782. doi: 10.1371/journal.pone.0049782 (PMC3506637; doi:10.1371/journal.pone.0049782)
Supplement: Table S2 — Association tests for haplotype blocks using Haploview for canine hip dysplasia and canine elbow dysplasia. The test statistics including χ2– and P-values (P) using 10,000 permutations for significantly associated haplotypes containing significantly associated SNPs from the general model analysis are shown. For these significantly associated SNPs, the SNP-ID and their chromosomal position are given. (DOC) [file pone.0049782.s002.doc]

**Table S2** **Association tests for haplotype blocks using Haploview for canine hip dysplasia and canine elbow dysplasia.** The test statistics including χ^2^– and P-values (P) using 10,000 permutations for significantly associated haplotypes containing significantly associated SNPs from the general model analysis are shown. For these significantly associated SNPs, the SNP-ID and their chromosomal position are given.

|  | Haplotype block | | | | | |
| --- | --- | --- | --- | --- | --- | --- |
| CFA | Start and end of the haplotype block in bp | Associated haplotype | χ^2^ | P | SNP-ID | Position (bp) |
| Canine hip dysplasia | | | | | | |
| 37 | 25,095,511-25,201,958 | AGCC | 10.6 | 0.023 | BICF2S23052396 | 25,095,511 |
| Canine elbow dysplasia | | | | | | |
| 11 | 18,803,152-19,071,837 | AGTTAAGCCCA | 15.1 | 0.011 | BICF2G630294653 | 18,913,755 |
| 11 | 19,114,139-19,132,047 | GT | 14.0 | 0.021 | BICF2G630294836 | 19,114,139 |
